# Quantifying the case for prevention: disease burden and cost-effective interventions in UK

**DOI:** 10.1093/pubmed/fdag028

**Published:** 2026-04-30

**Authors:** Lesley Owen, Matthew Taylor

**Affiliations:** Health Economics and Decision Modelling, Science, Evidence and Analytics Directorate, National Institute for Health and Care Excellence, 2 Redman Place, Stratford Cross, London, E20 1JQ, UK; York Health Economics Consortium, University of York, Level 2, Enterprise House, Innovation Way, York, North Yorkshire, YO10 5NQ, UK

**Keywords:** population-based and preventative services, communicable diseases, cost effectiveness

## Abstract

**Background:**

Modifiable risks—tobacco use, poor diet, alcohol consumption, physical inactivity and mental ill-health—drive substantial disease in UK. NICE evidence on preventive interventions targeting these risks and estimates of eligible populations and disease burden demonstrate the potential for prevention and reducing health inequalities.

**Methods:**

Deaths and DALYs from behavioural risks and mental health conditions were estimated using GBOD 2021 data. Prevalence by deprivation quintile came from national surveys. NICE guidelines were reviewed to identify cost effective preventive interventions.

**Results:**

In 2021, tobacco and dietary risks caused the highest mortality and disease burden: tobacco nearly 58 000 deaths and 1·4 million DALYs; diet 48 000 deaths and 1.05 million DALYs. Mental health had low mortality (5·7 deaths) but high DALYs (1·45 million), especially in younger people. Alcohol and inactivity had lower death counts and moderate DALY impacts. Most risks were more prevalent in deprived areas. NICE-recommended interventions such as smoking cessation, alcohol brief advice, obesity treatments, and psychological therapies, are relevant to millions, especially high-need groups.

**Conclusion:**

(Re-)investing in prevention and equitable strategies could substantially reduce disease burden and address inequalities, with the greatest gains likely to arise from sustained and targeted investment in populations and areas that have been left behind.

## Introduction

Prevention forms a significant part of the recently published NHS 10-year health plan which presents substantial opportunities to improve population health and reduce pressure on healthcare services.^1^ In 2021, non-communicable diseases (NCDs) were responsible for an estimated 424 320 deaths in UK and accounted for ~17.9 million disability adjusted life years (DALYs), highlighting the need for preventive action.^2^ The plan also emphasizes the importance of preventing mental ill health, particularly in children and young people, with nearly half of all mental health conditions developing before age 18. Conservative estimates suggest that £1 billion spent on public health could generate around 206 398 quality-adjusted life years (QALYs), more than three times the health gain achieved by the same investment in healthcare treatment, highlighting the exceptional value of prevention.^3^ A systematic review found median returns of 14·3 to 1·0 for public health interventions and 4·1 to 1·0 for local public health interventions.^4^

NHS England's own modelling further demonstrates the potential system gains from increased investment in prevention.^5^ Full rollout of comprehensive tobacco dependence treatment across hospitals and maternity services is estimated to avert nearly 42 000 admissions and around 150 000 bed-days in the first full year. Similarly, full-scale delivery of the NHS cardiovascular prevention programme (NHS Health Checks) could prevent ~1600 heart attacks, 650 strokes and 4000 new diabetes cases annually. Preventing even hundreds of such events each year translates into thousands of avoided admissions and substantial reductions in high-cost bed-day use, particularly in emergency cardiovascular care.

A key feature of the plan is its commitment to reducing health inequalities by prioritising investment in areas with the lowest healthy life expectancy such as working class and coastal communities. Through targeted funding and infrastructure, the plan aims to narrow the persistent gap between the most and least affluent regions.

Cost effective public health interventions are central to this long-term strategy, not only for improving population health, but also for addressing persistent disparities in health outcomes. Evidence-based interventions such as smoking cessation,^6^ obesity reduction^7^^,^^8^ and mental health support^9^ have been shown to reduce demand on acute services, thereby freeing up capacity for critical care.

Cardiovascular disease (CVD), which accounts for most NCD deaths,^10^ is among the conditions most strongly associated with inequalities. Individuals living in the most deprived areas of UK are nearly four times more likely to die prematurely from CVD than those in the least deprived areas.^11^

It is likely that interventions will have different impacts when applied to different populations (i.e. those in the most deprived areas compared with those in the least deprived areas). Different levels of awareness, uptake and adherence can all influence the effectiveness of an intervention. Furthermore, the benefits associated with a successful intervention can differ between populations (i.e. quality of life and life expectancy vary substantially between regions), and so the quantitative valuation of health benefits is not standardized across the whole country.^12^

Reducing preventable illness saves billions, both in direct NHS expenditure and broader societal costs. Despite being largely preventable, CVD alone costs the NHS an estimated £7·4 billion per year.^10^ Moreover, 40% of the burden on health services in UK is considered avoidable through action on modifiable risk factors such as smoking, obesity and physical inactivity.^13^ Health inequalities specifically are estimated to cost the NHS over £4·8 billion a year in hospitalisations.^14^

While the NHS Long Term Plan^1^ includes a commitment to shift spending towards prevention and out-of-hospital care, it does not specify a fixed proportion of the budget to be allocated. The 2023 Hewitt Review recommended increasing the share of NHS budgets at Integrated Care System level going towards prevention by at least 1% over the next 5 years from the current baseline of 4–5%.^15^

The cost effectiveness of public health interventions has long been subject to scrutiny, often with higher expectations to achieve short term cost savings compared to other areas of health expenditure.^16^ However, an analysis of public health interventions recommended by NICE showed that 75% are cost effective at the £20 000 per QALY gained threshold with 21% achieving net cost-savings.^17^

Most NCDs are attributable to four modifiable behavioural risk factors: tobacco use, physical inactivity, unhealthy diet and harmful alcohol consumption. In this analysis we synthesize NICE guideline evidence on prevention and estimate the size of the eligible populations to assess the potential population-level impact of interventions targeting these leading contributors to ill health in UK. Given the increasing recognition of mental health as a major component of disease burden, we also include relevant preventive interventions. For this purpose, the analysis focusses on individual-level approaches rather than broader population strategies. Additionally, we report the scale of morbidity and mortality attributable to these risk factors to contextualize the urgency and scope of preventive action.

## Methods

The Global Burden of Diseases, Injuries and Risk Factors Study (GBD) was used to estimate number of deaths and disability adjusted life years (DALYs) in UK in 2021 linked to key risk behaviours and mental health conditions.^18^ It is the most comprehensive international effort to quantify health trends across countries. DALYs were used as the primary metric to capture both premature mortality (years of life lost, YLL) and time spent in ill health (years of healthy life lost to disability, YLD). Estimates were restricted to NCDs and specified risk behaviours. Mental health conditions were assessed using mental disorders as the base as mental health-related deaths are typically classified outside the standard NCD categories. ‘All cause’ estimates were excluded to avoid overstating the burden estimates for individual risks.

Prevalence data on each risk factor, stratified by deprivation, were sourced from most recently available population-level surveys: the Health Survey for England (2022),^19^ the Office for National Statistics (2021)^20^ and the NHS England Adult Psychiatric Morbidity Survey 2023/4.^21^ These sources provide robust measures of behavioural risk and mental health indicators across deprivation groups using the Index of Multiple Deprivation (IMD). For the present analysis, deprivation was categorized into five quintiles with IMD1 representing the most deprived group and IMD5 the least deprived.

The National Institute for Health and Care Excellence guidance database was systematically searched using the key words ‘tobacco,’ ‘alcohol,’ ‘physical activity,’ ‘obesity’ and ‘mental.’ The term ‘mental’ was deliberately chosen to capture the broad range of relevant guidance, including those addressing mental wellbeing, mental health and mental illness, thereby minimising the risk of omitting pertinent documents. Seventeen guidelines were identified and a purposive sample with a clear preventive emphasis was selected using a simple manual screening process, with researcher judgement applied where terminology or guideline type varied. These guidelines were examined in detail to identify cost effective interventions, drawing on the associated NICE evidence reviews and economic modelling. Additionally, two technology appraisals included in the prevention guideline for overweight and obesity were included in the analysis.

Cost effective interventions were identified using the incremental cost-effectiveness ratio (ICER) which summarizes the economic value of an intervention relative to a comparator. The ICER is calculated by dividing the difference in cost (incremental cost) by the difference in Quality Adjusted Life Years (incremental QALYs). NICE considers interventions below £20 000 per QALY to be cost effective.

## Results

Table 1 presents the estimated number of deaths and DALYs associated with each risk category. Tobacco and dietary risks were associated with the highest numbers of deaths with 57 986 and 47 751 respectively. While mental health was linked to considerably fewer deaths (5·67) it contributed a considerable burden in terms of DALYs with an estimated 1·45 million, comparable to that for tobacco. Dietary risks accounted for 1·05 million DALYs. Among individuals aged under 20, mental health related mortality was low (0·43 deaths), however associated DALYs exceeded 200 000 indicating substantial years lived with disability. High alcohol use and low physical activity were linked to fewer deaths and a moderate burden of disease, with DALY estimates lower than those observed for tobacco, dietary risks and mental health. Across all risk categories, the ranges highlight uncertainty in modelled estimates, particularly for dietary risks.

**Table 1.** Burden of disease of key risk behaviour and mental health in UK in 2021.

Risk (all ages)	Number of Deaths^*^	*Range*	Number of DALYs	*Range*
**Tobacco**	57 987	*46 649–70 177*	1 397 612	*1·09 m–1·71 m*
**Dietary risks**	47 751	*10 811–73 878*	1 050 451	*259 146–1·59 m*
**High alcohol use**	14 547	*11 773–18 272*	562 015	*475 511–662 699*
**Low physical activity**	9272	*4011–14 797*	210 970	*93 540–323 680*
Mental health^*^	5·76	*5·14–6·35*	1 445 141	*1·07 m–1·89 m*
Mental health < 20 years^*^	0·43	*0·37–049*	201 701	*150 909–280 673*

Causes: Source GBOD^22^: NCDs base for risk behaviours

^*^Mental disorders base for mental health conditions

In England, the most prevalent mental health disorders fall under the category of common mental health conditions (CMHCs) which primarily include various forms of depression and anxiety. In 2023, one in five (20·2) adults had a CMHC. The most prevalent CMHCs were generalized anxiety disorder (7·5%), depression (3·8%), and the general category of CMHC-Not Otherwise Specified (8·6%).^21^

Table 2 shows the distribution of selected health related behaviours and mental health conditions across levels of deprivation in UK using the IMD, where IMD 1 is the most deprived quintile and IMD 5 is the least deprived. It shows a pronounced gradient in smoking prevalence, which is approximately three times higher in the most deprived areas compared to the least. Similar patterns are also seen for obesity, common mental health disorders and low physical activity. In contrast, being overweight is more common among less deprived groups. The prevalence of high alcohol consumption shows no discernible pattern by deprivation quintiles.

**Table 2.** Population prevalence (%) and health inequalities.

	Index of Multiple Deprivation (IMD1 = most deprived)					
	IMD 1	IMD 2	IMD 3	IMD 4	IMD 5	*Overall*
Current smokers (ONS 2021)^23^	22·0	15·0	12·0	9·0	7·0	*13·0*
Obese (HSE 2022)^25^	36·0	31·0	29·0	28·0	23·0	*28·0*
Overweight (HSE 2022)^24^	32·0	33·0	37·0	36·0	36·0	*35·0*
Mental Health (common mental health conditions) 2023–24^21^	26·2	22·9	19·1	17·9	16·0	*20·2*
High alcohol use (HSE 2022)^25^	11·0	14·0	13·0	12·0	12·0	*12·0*
Low physical activity (HSE 2022)^26^	30·0	25·0	22·0	21·0	18·0	*22·0*

Table 3 presents selected interventions assessed by NICE that have been deemed either cost effective or dominant (more effective and less costly). The table also includes estimates of the size of the population eligible for the interventions, highlighting the substantial potential for reducing ill health through these evidence-based prevention interventions.

**Table 3.** Cost-effective public health interventions and estimated eligible populations in UK.

Risk Factor/Condition	Intervention examples	Cost-Effectiveness (ICER)	Estimated Eligible Population (UK)
Smoking^27^	NRT, varenicline, bupropion, CBT, incentives, opt-out, tailored interventions	Maximum of £6875/QALY, with most interventions being dominant	6 million adults; 2·6 million CMD, 38 883 pregnant women; 143 435 adults with severe mental illness
Alcohol use^28^	Screening and brief interventions, primary care	£5865–£21 430/QALY, with most interventions being dominant	10 million adults
Poor diet/Obesity^29–32^	Behavioural, physical activity, semaglutide, tirzepatide	£6317—£21 372/QALY, with some interventions dominant	28 million adults overweight or obese
Physical inactivity^28^^,^^33^	Brief advice, walking groups, workplace programmes	£495—£2754/QALY, with some interventions dominant	17 million adults
Mental Ill-health^34^^,^^35^	Group CBT, computerised CBT, behavioural activation, pharmacological treatments	£541–£10 905/QALY, with some interventions dominant	7·5 million adults; 1·45 million with depression; 2·6 million with anxiety
Mental Wellbeing^28^^,^^33^	Friendship programmes, walking, digital literacy for older adults	Maximum £15 962/QALY with some interventions being dominant	10·8 million aged 65+

^*^Base = All figures refer specifically to smokers within that group e.g. 6 million adult smokers

ICER = Incremental cost-effectiveness ratio; NRT = Nicotine replacement therapy; CBT = cognitive behavioural therapy; QALY = quality-adjusted life year; CMD = Common mental disorders

### Smoking

In 2023, there were 6 million current smokers in UK. Of these, 2·6 million smokers had a common mental disorder,143435 had a serious mental health condition and 38 883 were pregnant women.

In the general population, multiple interventions were found to be dominant (more effective and less costly) compared with usual care. For pregnant women, two of the three interventions assessed—financial incentives and opt-out referral—were also dominant. The third intervention—long and short acting nicotine replacement therapy—was cost effective with an ICER of £5281 per QALY gained. For individuals with severe mental illness interventions were also cost effective. Bespoke interventions resulted in an ICER of £3145/QALY and integrated care was £6875/QALY.

### Alcohol use

Brief interventions and screening in primary care are mostly dominant but ICERs can also range from £5865–£21 430/QALY depending on who delivers the intervention, the setting, and the specific audit tool used for assessment. These interventions are relevant to 10 million adults in UK who consume alcohol above the Government’s low-risk drinking guidelines.

### Poor diet and obesity

Behavioural, physical activity and diet interventions, as well as pharmacological treatments such as semaglutide and tirzepatide, are all deemed cost-effective, with ICERs ranging from £6317 to £21 372 per QALY depending on the target population. These interventions are relevant to an estimated 28 million adults in UK who are overweight or obese.

### Physical inactivity

Interventions such as brief advice, walking groups, and workplace programmes demonstrate strong cost-effectiveness, with ICERs ranging from £495 to £2754 per QALY when compared against usual care (which may include ‘no intervention’). Brief interventions are particularly effective for older adults and those with chronic conditions, with some assessed as dominant in terms of cost effectiveness. In UK, the eligible population is estimated to be 17 million adults who do not meet the recommended physical activity levels for maintaining good health.

### Mental ill-health

Interventions for common mental disorders—including psychological, psychosocial, pharmacological and combination treatments—show variable cost-effectiveness, depending on delivery model and target population. These interventions are relevant to ~7·5 million adults.

### Depression (less severe)

Group and individual cognitive behavioural therapy (CBT), behavioural activation, group exercise and pharmacological treatments yield ICERs ranging from £845 to £10 905 per QALY. These are applicable to 1.45 million adults with less severe depression.

### Anxiety

Psychological interventions including low intensity and computerized CBT (CCBT) as well as pharmacological treatments such as sertraline are highly cost-effective. CCBT yields an ICER of £541 per QALY and sertraline is classified as a dominant intervention. These interventions are relevant to an estimated 2.6 million adults in England living with anxiety disorders.

### Mental wellbeing in older adults

Community-based walking, friendship programmes and computer and internet training demonstrate cost-effectiveness with ICERs ranging from £7372 to £15 962 per QALY. Some interventions are classified as dominant. They are applicable to 10·8 million adults aged 65 and over, highlighting their potential to support healthy ageing and social inclusion.

## Discussion

### Main finding of this study

This study demonstrates that a substantial burden of disease in England is driven by modifiable risk factors such as tobacco use, poor diet, alcohol consumption, physical inactivity and mental ill-health. Collectively these risks account for over 130 000 deaths and more than 5 million DALYs annually (GBOD). These risks are socio-economically patterned, disproportionately affecting deprived communities and contributing to persistent health inequalities. They also place significant strain on the NHS and wider public services.

The analysis finds that many public health and mental health interventions identified in NICE guidelines are not only highly cost effective when compared against usual care (which, in some cases, is doing nothing) but, in some cases, are dominant, delivering greater health benefits at lower total costs than usual care. These findings strengthen the case for upstream investment in prevention, particularly when targeted in areas of higher deprivation. Preventive interventions can improve population health, reduce avoidable mortality, and narrow inequalities within the English healthcare system. Alongside the health gains, such interventions have the potential to deliver significant cost savings across health and social care by reducing the prevalence of illness and premature mortality.

### What is already known on this topic

Previous evaluations by NICE have shown that the majority of public health interventions are cost effective. The most recent analysis found that 75% of public health interventions assessed were cost-effective at the £20 000 per QALY threshold with 21% resulting in cost-savings.^17^

It is well established that modifiable behavioural risks including smoking, alcohol use, poor diet, and physical inactivity are leading contributors to the burden of disease and are strongly influenced by socio-economic factors.

The Health Foundation emphasizes that population-level interventions, particularly those that shape the environments in which people live such as changes to food systems and urban design are more likely to be both effective and equitable.^36^ In England, smoke-free legislation, tobacco taxation, food and menu labelling regulations, and national sugar and salt reformulation programme provide examples of these measures which help create healthier environments. However, individual-level approaches remain important within comprehensive prevention strategies. Many of the cost-effective interventions in this analysis, such as behavioural support and psychological therapies can be scaled and targeted to high-need groups, contributing to improved health outcomes and reduced inequalities.

### What this study adds

This study synthesizes a selection of cost-effective interventions from NICE guidelines addressing England's principal contributors to morbidity and mortality. By linking these interventions with burden of disease and deprivation data, it identifies where preventive action is likely to deliver the greatest population health gains and contribute to reducing health inequalities, supporting a more targeted and equitable approach to public health investment.

The study also places these findings within the strategic priorities of the NHS 10-year plan, which advocates a shift from treatment to prevention through integrated, community-based care. It highlights the pivotal role of Integrated Care Systems and local authorities in driving this transformation.

Recent national evidence further strengthens the case for targeted preventive investment. The Chief Medical Officer’s 2025 report^37^ highlights substantial and persistent variation in preventive service delivery across geography, socioeconomic status, age, gender and ethnicity, with some populations benefitting far less from recent improvements in health. At the same time, disinvestment in key preventive services, such as smoking cessation where several NHS trusts have begun decommissioning services and 83% face funding uncertainty, disproportionately affects those groups with the poorest outcomes.^38^ These patterns suggest that the greatest marginal gains are likely to come not only from sustaining investment in effective preventive interventions, but from directing additional investment toward the populations and places that have been consistently left behind.

The intersection of high disease burden, strong cost-effectiveness, and social patterning of risk presents a compelling case for sustained investment in prevention. When implemented at scale, particularly in areas of greatest need, these interventions can improve population health, reduce avoidable mortality, and narrow inequalities within the English health care system. They may also deliver substantial savings across health and social care expenditure by reducing the prevalence of illness and premature mortality.

### Limitations

Estimates of disease burden are inherently uncertain, largely due to limitations in data quality, coverage, and modelling assumptions. The World Health Organization recommends the use of uncertainty intervals, such as ranges for deaths and disability-adjusted life years (DALYs), to quantify this. However, these ranges may not fully capture all sources of variation and should be interpreted with caution, particularly given the challenges of attributing outcomes to specific risk factors.

National surveys, including those conducted by the Office for National Statistics (ONS) and Health Survey England (HSE), are commonly used to estimate the size of the eligible populations. However, these surveys are subject to sampling error and non-response bias, especially among marginalized or socioeconomically deprived groups. Moreover, reliance on self-reported data introduces further limitations, such as underreporting of behaviours like smoking and alcohol consumption. Importantly, identifying eligibility does not equate to predicting engagement and adherence with interventions.

QALYs, while widely adopted for health economic evaluations, may undervalue preventive interventions due to their longer time horizons and wider benefits. Standard modelling approaches, such as those used by NICE, typically assume average effects which may obscure local variations and mask the impact on health inequalities. This is particularly concerning in areas facing disproportionate health burdens, where the impact of prevention strategies may yield substantially different marginal gains, depending on the level of awareness, uptake, adherence and long-term outcomes.

While national cost-effectiveness thresholds provide a consistent framework for decision-making, they may not fully reflect opportunity costs in areas with distinct local priorities or resource constraints. In some cases, introducing a new policy may displace existing interventions, potentially causing more harm than benefit. As such, it is important that decision makers understand *which* interventions are likely to be displaced and the implications for population health. As highlighted earlier, conservative estimates suggest that £1 billion spent on public health generates over three times the health benefit of the same investment in healthcare treatment.

## Data Availability

All data used in this study are publicly available. GBD 2021 estimates were accessed via the IHME data portal. Prevalence data were obtained from the Health Survey for England (2022), Office for National Statistics (2021) datasets, and the NHS England Adult Psychiatric Morbidity Survey (2023/24). NICE guidelines and evidence reviews were accessed through the NICE website. No proprietary data were used, and all sources are cited in the manuscript.

## Notes

**Lesley Owen**, Health Economics Adviser, NICE

**Matthew Taylor**, Chief Executive Officer (CEO) of the York Health Economics Consortium (YHEC)
